# Prevalence, Antibiotic Susceptibility and Diversity of *Vibrio parahaemolyticus* Isolates in Seafood from South China

**DOI:** 10.3389/fmicb.2017.02566

**Published:** 2017-12-20

**Authors:** Ying Yang, Jiafang Xie, Hua Li, Shuwen Tan, Yanfeng Chen, Hui Yu

**Affiliations:** School of Life Science and Engineering, Foshan University, Foshan, China

**Keywords:** *Vibrio parahaemolyticus*, prevalence, antibiotic resistance, virulence gene, MLST

## Abstract

*Vibrio parahaemolyticus* is a leading cause of foodborne infections in China and a threat to human health worldwide. The main objective of this study is to determine the prevalence and characteristic of *V. parahaemolyticus* isolates in fish, oyster and shrimp samples from the South China domestic consumer market. To accomplish this, we examined 504 seafood samples from 11 provinces of China. The prevalence rates were 9.38, 30.36, and 25.60%, respectively. In summer (33.33%), the prevalence of *V. parahaemolyticus* was more common than that detected in the winter (14.01%). In addition, we identified 98 *V. parahaemolyticus* strains. The antimicrobial resistance trends of our seafood isolates to 15 antimicrobial agents revealed that major isolates were resistant to ampicillin (79.59%). Furthermore, 68.38% of the isolates were identified as being multidrug resistance. The prevalence of *tdh* or *trh* genes among the isolates was 8.16 and 12.24%, respectively. ERIC-PCR and multilocus sequence typing (MLST) results enabled classification of the isolates (*n* = 98) into different clusters, revealing genetic variation and relatedness among the isolates. Thus, our findings demonstrate the prevalence of *V. parahaemolyticus* in a variety of common seafood consumed domestically in China and provides insights into the dissemination of antibiotic-resistant strains, which should improve our microbiological risk assessment knowledge associated with *V. parahaemolyticus* in seafoods.

## Introduction

Foodborne disease remains a threat to public health worldwide. *Vibrio parahaemolyticus* is a halophilic Gram-negative bacterium that is commonly associated with food-borne gastroenteritis. This bacterium has been found in seawater, marine fish, shellfish, etc. (Fujino et al., 1953; Joseph et al., 1982; Kang et al., 2016) and causes outbreaks worldwide. In China, *V. parahaemolyticus* is also an important cause of food poisoning associated with consumption of seafood (Yu et al., 2015; Tan et al., 2017). It was also reported that the population levels of *V. parahaemolyticus* tends to show strong seasonal trends (Shen et al., 2010). Consequently, foodborne outbreaks caused by *V. parahaemolyticus* typically show a seasonal difference, peaking in the warmer months of the United States (Daniels et al., 2000). However, to date, the presence and contamination levels of *V. parahaemolyticus* in seafood from different seasons has received less attention, and little information is available among various types of samples.

As a result of the excessive use of antibiotics in human and aquaculture systems, some antibiotics have no longer been effective in controlling pathogens infections during the past several decades (Elmahdi et al., 2016). Variation in antibiotic resistance patterns among *V. parahaemolyticus* strains isolated from different countries and sources has been observed (Odeyemi and Stratev, 2016). Recent studies regarding a *V. parahaemolyticus* isolate demonstrated resistance to certain common antibiotics such as ampicillin and kanamycin (Lopatek et al., 2015; Xie et al., 2015). The frequent occurrence of multi-drug resistance in *V. parahaemolyticus* is among the most important of public health concerns (Xie et al., 2017). In order to improve yield and treatment of infectious diseases in farm products, increase levels of antibiotics in animal feed and water is a common behavior (Letchumanan et al., 2015a). Therefore, it is very important to set up a monitoring system to research on antimicrobial-resistance trends.

An important virulence genes, *toxR* is involved in the regulation of many genes in *V. parahaemolyticus* (Lin et al., 1993). The pathogenicity of *V. parahaemolyticus* is strongly correlated with the production of either thermostable direct hemolysin (TDH), TDH related hemolysin (TRH), or both (Honda and Iida, 1993; Mala et al., 2016). *V. parahaemolyticus* strains isolated from diarrheal patients produce thermostable TDH which is a toxin with several biological properties, including hemolytic activity, enterotoxicity and cytotoxicity, TDH is also present in most of the Kanagawa phenomenon (KP)-positive clinical strains. TRH is believed to act similarly to TDH. (Takahashi et al., 2000; Matsuda et al., 2010; Leoni et al., 2016). Presently, PCR-based methods is useful in identified of *tdh* and *trh* gene in *V. parahaemolyticus* isolates (Shirai et al., 1990).

The utility of molecular techniques has already been established in epidemiological studies of *V. parahaemolyticus* infections, such as pulsed-field gel electrophoresis (PFGE) (Marshall et al., 1999) and random amplified polymorphic DNA (RAPD) analysis (Yang et al., 2008). Enterobacterial repetitive intergenic consensus sequence PCR (ERIC-PCR) is an easy, fast, and relatively cheap method that is comparable to PFGE in terms of its discriminatory power and reproducibility, It is also superior to other PCR-based fingerprinting techniques (Wong and Lin, 2001; Jun et al., 2012). Multilocus sequence typing (MLST) is based on sequence analysis of housekeeping (HK) genes and is a useful method to trace the global epidemiology of bacterial pathogens. MLST, which is sequence based, provides a clear determination of the genetic features of strains that is consistent from one laboratory to another. Previous MLST studies of *V. parahaemolyticus* isolates have provided a better understanding of the genetic relatedness within these bacteria. These studies also revealed the relative evolutionary importance of mutations and lateral gene transfer events among these strains (Gonzálezescalona et al., 2008; Banerjee et al., 2014).

Overall, *V. parahaemolyticus* strains are often isolated from fish, shrimp and other types of seafoods. Recently, *V. parahaemolyticus* was also found in ready to eat food (Cho et al., 2016; Mala et al., 2016). In the Zhejiang province of China alone, there were 71 outbreaks caused by *V. parahaemolyticus* resulting in 933 illnesses and 117 hospitalizations from 2010 to 2014. In our study, we analyzed 224 fish, 112 oyster, and 168 shrimp samples. In China, seafood is very popular and higher consumption is correlated with an increase in the overall standard of living in the population. Our study aimed to find the differences in the seasonal prevalence of *V. parahaemolyticus* in all provinces of South China. We next characterized the prevalence all of the identified isolates to determine their genetic relatedness by phenotyping and genotyping methods. We attempted to confirm the virulence and antibiotic resistance trends. We also performed ERIC and MLST typing of the isolated strains, which revealed the molecular diversity of the *V. parahaemolyticus* isolates. These findings serve as baseline values that support the establishment of a national seafood surveillance system to ensure food safety in China.

## Materials and Methods

### Sample Collection

**FIGURE 1:**
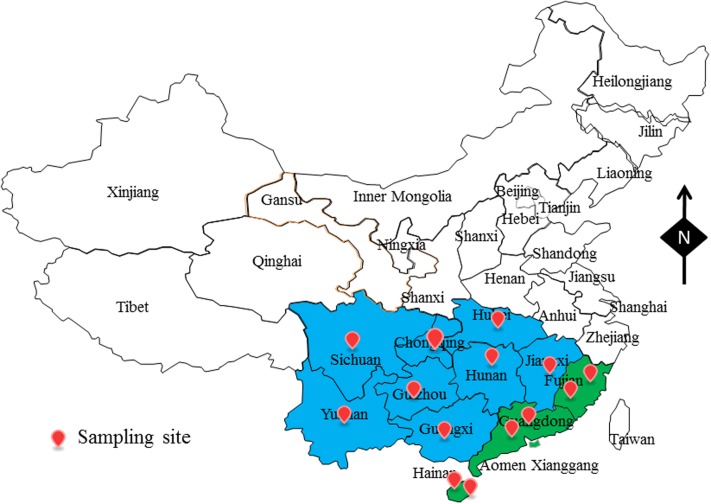
The sampling site of foods sample in South China.

From July 2015 to July 2017, a total of 504 aquatic products samples, including 224 fish samples, 112 oyster samples and 168 shrimp samples were collected from retail markets from 14 different cities in China, belonging to 11 provinces (**Figure 1**). In these regions, the climate is cold from September to March (winter), and it is hot from March to September (summer). From each city, we collected eight fish samples, four oyster samples, and six shrimp samples (from four different retail markets) during both summer and winter months. We put the samples in sterile sealed plastic bags and sent them to the lab in an ice box (4°C). Samples were analyzed immediately thereafter.

### Detection and Enumeration of *V. parahaemolyticus*

The prevalence and bacterial load of *V. parahaemolyticus* in the samples were tested using the most probable number (MPN) method according to the National Food Safety Standards of China (GB4789.7-2013); this method was reported previously by Xie et al. (2016). In brief, we first weighed and homogenized 25 g of each sample. Then, 225 mL of alkaline peptone water (APW) with 3% NaCl was added; 3 × 1 mL of each sample dilution was inoculated into 9 mL of APW (3% NaCl) when prepared up to a 1:1000 dilution. Samples were incubated at 37°C for 18 h. After that, we used an inoculation loop to streaked the solution onto thiosulfate-citrate-bile salts-sucrose (TCBS) agar plates and incubated them at 37°C for 24 h. The r green or blue green colonies of 2–3 mm in diameter were presumed to be *V. parahaemolyticus* isolates, three to five colonies (if present) were chose from each plate, streaked onto Chromogenic Vibrio Medium and culture at 37°C for 24 h. One (if present) colony (mauve) from each Chromogenic Vibrio Medium plate was selected for further identification tests, such as Gram staining, NaCl tolerance tests, oxidase activity and analysis with API 20E diagnostic strips (BioMerieux, Marcy-l’Étoile, France). The pollution result in the samples were determined using an MPN table. The levels of contamination in the samples were determined using an MPN table.

### Antimicrobial Susceptibility Testing

The susceptibility of the *V. parahaemolyticus* isolates to antibiotics was examined by the disk-diffusion method, according to the guidelines of the Clinical and Laboratory Standards Institute (CLSI, 2012; Miller et al., 2014). Muller – Hinton agar and a panel of 15 antibiotics disks were selected for the resistance analysis. The 15 common antimicrobials used in this study belonged to six classes and were as follows: β-lactam [ampicillin: AMP (10 μg); piperacillin: PRL (20 μg); cefotaxime: CTX (30 μg); cefoxitin: FOX (30 μg); cephazolin: KZ (30 μg); imipenem: IPM (20 μg); meropenem: MEM (20 μg)], aminoglycoside [gentamicin: CN (10 μg); kanamycin: K (30 μg); streptomycin: S (10 μg)], tetracycline [tetracycline: TET (30 μg)], quinolone [ciprofloxacin:CIP (5 μg); levofloxacin: LEV (20 μg)], sulfonamides [trimethoprim-sulfamethoxazole: SXT (25 μg)], chloramphenicol [chloramphenicol:C (30 μg)]. Following the methods of the CLSI, sensitive (S), intermediate (I), or resistant (R) classification system was used.

### Detection of Virulence Genes: *toxR*, *tdh*, and *trh*

According to the manufacturer’s instructions, genomic DNA was extracted from each *V. parahaemolyticus* isolate using a bacterial DNA extraction kit (Sangon, Shanghai, China).

The *toxR* gene is important and appears to be well conserved among *V. parahaemolyticus* isolates. The primers were as follows F: GTCTTCTGACGCAATCGTTG, R: ATACGAGTGGTTGCTGTCATG (Kim et al., 1999). The *tdh* and *trh* genes detection was executed as previously reported (West et al., 2013). The following primers were used: Tdh-F:CTGTCCCTTTTCCTGCCCCCG, Tdh-R:AGCCAGACACCGCTGCCATTG;Trh-F:ACCTTTTCCTTCTCCWGGKTCSG,Trh-F:CCGCTCTCATATGCYTCGACAKT). All the oligonucleotide primers were synthesized by Sangon Biotech (Shanghai, China). The PCR reaction system contained DNA template (1 μL), 0.5 μM of each primer, 12.5 μL of :2 × PCR mix (Qiagen), and ddH_2_O (9.5 μL). The amplified thermal-cycling program was set with the following conditions: denaturation at 95°C (5 min); 40 cycles of 95°C for 1 min, 62°C for 1 min, and 72°C for 1 min; and a final extension step of 72°C for 5 min. PCR amplicons were electrophoresed on 2.0% (wt/vol) agarose gels containing GoldView. The images were captured digitally and analyzed using a gel imaging system. *V. parahaemolyticus* strains ATCC33847 (*tdh*+) and ATCC17802 (*trh*+) were used as positive controls, and distilled water was used as a negative control.

### ERIC-PCR Analysis

ERIC-PCR analysis was performed on the *V. parahaemolyticus* isolates using a previously reported method (Chen et al., 2012; Xie et al., 2015). A pair of primers, ERIC - F (5-ATGTAAGCTCCTGGGGATTCAC-3) and ERIC -R (5-AAGTAAGTGACTGGGGTGAGCG-3) were used. ERIC-PCR typing was performed as follows: 1 μl of genomic DNA (100 ng) was added to a master mixture that contained 0.6 μmol/L of each primer, 12.5 μL of 2 × Long Taq mix (Dongsheng Biotech, Guangzhou, China), and ddH_2_O to a total volume of 25 μL per reaction. After an initial denaturation at 95°C for 5 min, 35 cycles of amplification were performed under the following conditions: denaturation at 94°C for 45 s, annealing 52°C for 1 min and extension at 72°C for 3 min followed by a final extension step at 72°C for 10 min. The amplicons were electrophoresed on 2.0% (wt/vol) agarose gels containing GoldView. The images were captured digitally in TIFF file format for further analysis.

### MLST Analysis

The *V. parahaemolyticus* MLST website and database^1^ shows the MLST analysis procedure (Jolley et al., 2004). Seven genes were selected (*recA*, *gyrB*, *dnaE*, *dtdS*, *pntA*, *pyrC*, and *tnaA*). The amplified thermal-cycling program was designed with the following conditions: denaturation at 95°C (5 min); 30 cycles of 95°C for 1 min, 58°C for 1 min, and 72°C for 1 min; and a final extension step of 72°C for 10 min. A BGI instrument (Shenzhen, China) was used to sequence the PCR amplicons. The assigning allele numbers and defined sequence types (STs)^1^. BioEdit was used to determine the alignments of these sequences.

### Statistical Analysis

The size of each band in the ERIC patterns was determined and the data were coded as 0 (absent) or 1 (present). Cluster analysis was performed with NTSYS-pc (version 2.10), a numerical taxonomy and multivariate analysis software package (Rohlf, 2000), based on Dice’s similarity coefficient (SD), with a 1% position tolerance and the unweighted-pair group method with arithmetic averages (UPGMA). The evolution tree of the concatenated sequences of the seven loci (MLST) was built based on the method involving the Kimura-2-parameter in Mega 6.0 (Tamura et al., 2013).

## Results

### Prevalence and Level of *V. parahaemolyticus*

Of the 504 seafood samples that we tested, 98 (19.44%) were identified as *V. parahaemolyticus* positive. This included 21 (9.38%) of the 224 fish samples, 34 (30.36%) of the 112 oyster samples and 43 (25.60%) of the 168 shrimp samples. These results are shown in **Table 1**. Overall, the degree of *V. parahaemolyticus* contamination in these samples varied from 1.50 to 1000 MPN/g. Most of the positive samples had a level of 3 to 10 MPN/g (62/98, 63.27%). Moreover, 19.39% (19/98) of the positive samples exceeded 100 MPN/g, and 17 samples were below 3 MPN/g. In total, 98 *V. parahaemolyticus* isolates were confirmed (Supplementary Table S1).

**Table 1:** Prevalence of *Vibrio parahaemolyticus* in food samples from South China.

Samples	Number of samples analyzed	Number of samples positive (%)	Number of samples containing the pathogen		
			<3 (MPN/g)	3–100 (MPN/g)	>100–10^3^ (MPN/g)
Fish	224	21 (9.38)	3	13	5
Oyster	112	34 (30.36)	8	16	10
Shrimp	168	43 (25.60)	6	33	4
Total	504	98 (19.44)	17	62	19

Considering the influence of the season, the number of positive summer samples was much higher than the number of positive winter samples (**Table 2**). The isolation rate of *V. parahaemolyticus* in summer reached 33.33%, whereas it was 14.01% in the winter. Moreover, the *V. parahaemolyticus* population differed significantly for samples collected during the winter and summer. Densities among samples collected in the summer varied less, and the contamination level was higher than that observed in the winter samples. In summer, the mean levels of *V. parahaemolyticus* in samples were 99.08 MPN/g, which is higher than that observed in the winter (22.13 MPN/g). A total of 14 samples with densities above 100 MPN/g were detected.

**Table 2:** Prevalence of *Vibrio parahaemolyticus* during different seasons.

Samples	Number of samples analyzed	Number of samples positive (%)	Number of samples containing the pathogen		
			<3 (MPN/g)	3–100 (MPN/g)	>100–10^3^ (MPN/g)
Summer	207	69 (33.33)	9	46	14
Winter	207	29 (14.01)	8	16	5
Total	905	98 (19.44)	17	62	19

### Antimicrobial Susceptibility of the *V. parahaemolyticus* Isolates

Isolates of *V. parahaemolyticus* were tested for antibiotic susceptibility, the resistance patterns to 15 antibiotics are shown in **Table 3**. The isolates were most resistant to ampicillin, with resistance and intermediate rates of 79.59 and 12.24%, respectively. In addition, the isolates exhibited relatively high resistance rates, of 68.37, 39.80, and 39.80%, for streptomycin, cefazolin, and kanamycin, respectively. Fortunately, all the examined isolates were susceptible to cefotaxime, cefoxitin, imipenem and meropenem. Among the remaining tested antibiotics, the isolates were partially susceptible to piperacillin, ciprofloxacin, levofloxacin, or chloramphenicol. However, among all the isolates, there were multidrug-resistant isolates (Vps15) showing resistance to seven antibiotics and some strains showing resistance to five antibiotics. In addition, 68.38% (67/98) of the isolates were resistance to more than three antibiotics (Supplementary Table S1).

**Table 3:** Antimicrobial resistance profiles of *Vibrio parahaemolyticus* isolates.

Antimicrobial agents	*Vibrio parahaemolyticus* (*n* = 98)		
	Number	Number	Number
	(%) of R	(%) of I	(%) of S
Ampicillin (AMP)	78 (79.59)	12 (12.24)	8 (8.16)
Piperacillin (PRL)	8 (8.16)	1 (1.02)	89 (90.82)
Cefotaxime (CTX)	0 (0.00)	2 (2.04)	96 (97.96)
Cefoxitin (FOX)	0 (0.00)	1 (1.02)	97 (98.98)
Cefazolin (KZ)	39 (39.80)	18 (18.37)	41 (41.84)
Imipenem (IPM)	0 (0.00)	2 (2.04)	96 (97.96)
Meropenem (MEM)	0 (0.00)	0 (0.00)	98 (100.00)
Gentamicin (CN)	17 (17.35)	20 (20.41)	61 (62.24)
Kanamycin (K)	39 (39.80)	24 (24.49)	35 (35.71)
Streptomycin (S)	67 (68.37)	26 (26.53)	5 (5.10)
Tetracycline (TE)	13 (13.27)	8 (8.16)	77 (78.57)
Ciprofloxacin (CIP)	5 (5.10)	2 (2.04)	91 (92.86)
Levofloxacin (LEV)	1 (1.02)	5 (5.10)	92 (93.88)
Trimethoprim-sulfamethoxazole (SXT)	9 (9.18)	12 (12.24)	77 (78.57)
Chloramphenicol (C)	2 (2.04)	1 (1.02)	95 (96.94)

### The *toxR*, *tdh*, and *trh* Genes Test

To detect pathogenic isolates, all of the 98 *V. parahaemolyticus* isolates were examined for the presence of the *toxR*, *trh*, and *tdh* genes. This analysis is summarized in **Table 4**. All of the isolates were positive for *tox R*. Among them, 8.16% (8/98) and 12.24% (12/98) of the *V. parahaemolyticus* strains carried the *tdh*, or *trh* gene, respectively; in contrast, none of the isolates harbored both the *tdh* and *trh* genes. For the *tdh* -positive strains, two were found in a fish source, two were identified from oysters, and three were found from shrimp. Of the *trh*-positive isolates, five samples were from fish, four samples were from oyster, and three samples were from shrimp.

**Table 4:** Distribution of the *tox R*, *tdh*, and *trh* genes in *V. parahaemolyticus* isolates.

Sample category	Number of isolates	*tox R*-positive (%)	*tdh*- positive (%)	*trh*- positive (%)
Fish	21	100 (21/21)	2	5
Oyster	34	100 (34/34)	2	4
Shrimp	43	100 (43/43)	4	3
Total	98	100 (98/98)	8.16 (8/98)	12.24 (12/98)

### ERIC-PCR and MLST

**FIGURE 2:**
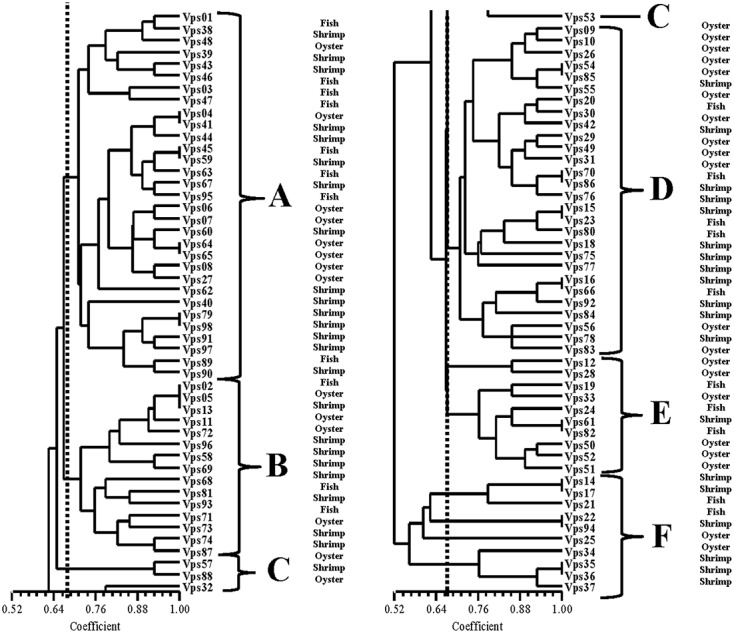
ERIC-PCR DNA fingerprint analysis of *V. parahaemolyticus* isolates.

**Figure 2** shows the results from ERIC-PCR analysis of the 98 isolates. There were 5 to10 amplification bands, with sizes ranging between 100 bp to about 6000 bp. Analysis of the ERIC-PCR patterns revealed that those strains could be divided into six clusters, A, B, C, D, E, and F at a relative similarity coefficient of 0.67. The isolates proved to be very diverse genetically. Most isolates were distributed among the A and D clusters. However, we did not find a relationship between the different sample types and the ERIC-PCR clusters

**FIGURE 3:**
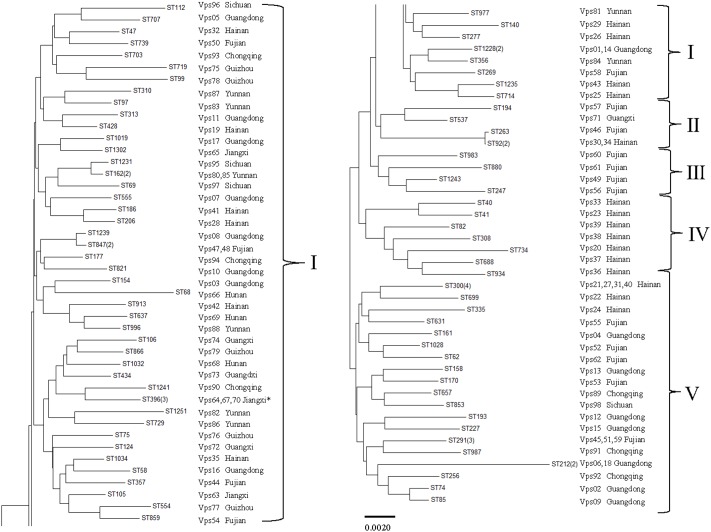
Multilocus sequence typing (MLST) minimum evolution tree of *V. parahaemolyticus* isolates (“^∗^” stand for “Jiangxi, Hunan, Guangxi”).

The isolates were analyzed by MLST using the sequences generated from the internal fragments of the seven HK genes. The alleles numbers and STs were assigned according to a database created for *V. parahaemolyticus* isolates upon submitting the sequence results. A total of 86 STs were observed among the 98 isolates. The numbers of alleles observed for each MLST locus in this study showed statistics as follows: *dna E*: 61; *gyr B*: 70; *rec A*: 62; *dtd S*: 57; *pnt A*: 43; *pyr C*: 60; and *tna A*: 42. The haplotype diversity was 0.987. A minimum evolutionary tree was constructed using the concatenated sequences of each allele. The MLST results divided the *V. parahaemolyticus* isolates into five clusters (designated as I, II, III, IV, and V). Most isolates were distributed in cluster I. Interestingly, **Figure 3** shows that all the isolates in cluster III were from Fujian, whereas, cluster IV contained the *V. parahaemolyticus* isolates from Hainan. In cluster V, most of the isolates were from three coastal cities. For strain Vps18 and Vps06 (from Guangdong), the ST type (ST212) showed a greater distance on the evolutionary tree.

## Discussion

According to epidemiologic reports, *V. parahaemolyticus* is a major cause of bacterial infections associated with the consumption of raw or undercooked aquatic products including different kinds of shellfish and fish (Tan et al., 2017). Our results show that the prevalence of *V. parahaemolyticus* was higher in oyster samples (30.36%) than in the other seafood samples. This prevalence is also lower than that found in Germany (41.8%) (Jones et al., 2012). The prevalence of *V. parahaemolyticus* isolates detected off the eastern coast of China was also reported to be higher than that observed in our study (Zhao et al., 2011). The reason for this discrepancy maybe that most of the sampled cities were inland, with much fewer kinds of seafood. Consistent with this hypothesis, the prevalence of this bacteria in coastal cities (Guangdong, Hainan, and Fujian) was higher (Supplementary Table S1).

Notably, the prevalence of *V. parahaemolyticus* in summer (33.33%) was higher than that in winter (14.01%). Our results are in accordance with previous reports that *V. parahaemolyticus* outbreaks typically show a seasonal pattern, peaking in the warmer months (Daniels et al., 2000; Chen et al., 2017), and that *V. parahaemolyticus* isolates are cold sensitive, thus, lower temperature in winter may limit or reduce their growth (Su and Liu, 2007). As our results were not only obtained from samples collected from a majority of regions in South China, but also from different seasons and kinds of seafood, the data can be useful for determining optimal temperature values for control of *V. parahaemolyticus* contamination (World Health Organization, 2014).

As there is an increase in the number of resistance genes and the spread of antimicrobial-resistant *V. parahaemolyticus* isolates worldwide, the misuse and overuse of antibiotics are considered the most important factors (Yano et al., 2014; Tan et al., 2016). In the Hunan province of China, the average utilization rate of antibiotics reached 64.56% and more than 50 antibiotics were available for sale (Daimei and Dai, 2016). Susceptibility tests show that isolates *V. parahaemolyticus* in South China appear to a high level of resistance to ampicillin. This result is similar to a report by Letchumanan et al. (2015b) in which 82% of the isolates from shrimp samples were also resistant to ampicillin. In addition, many isolates were resistant to streptomycin (68.37%) and kanamycin (39.80%). Similarly, previous studies have suggested that resistance to these antibiotics is common in *V. parahaemolyticus* isolates (Elexson et al., 2014; Shaw et al., 2014). Susceptibility profiles to antibiotic classes such as cefotaxime, cefoxitin, imipenem, and meropenem were examined. Notably, we found some isolates showing resistance to chloramphenicol, tetracycline or ciprofloxacin, which are first-line drugs used in clinical treatment (Ottaviani et al., 2013; Elmahdi et al., 2016). In this work, 68.38% of the strains were multi-drug resistant and some even showed resistance to seven antibiotics. This ratio is higher than that detected in previous reports (Letchumanan et al., 2015a; Xu et al., 2016). In general, evaluating variations in the antimicrobial susceptibility trends of *V. parahaemolyticus* strains is important give that infection emergence of microbial resistance to multiple drugs is a serious clinical problem.

The *toxR* gene, which is involved in the regulation of many other genes, was ubiquitous in *V. parahaemolyticus* isolates. As we know, not all *V. parahaemolyticus* strains are pathogenic in humans; however, the products of the hemolysin genes (*tdh* and *trh*) are believed to rapidly induce inflammatory gastroenteritis and are often detected in clinical strains (Mahoney et al., 2010; Raghunath, 2015). Thus, detection of the hemolysin genes could be an important way to infer the virulence potential of food isolates. In our study, up to 8.16 and 12.24% of the strains were *tdh* or *trh* positive. Our findings are similar to those of previous studies on environmental *V. parahaemolyticus* isolates (Gutierrez West et al., 2013; Letchumanan et al., 2015a). The distributions and evolution of *tdh*+ and/or *trh*+ strains may differ depending on sample source, geographical region, and other environmental factors (Wilson and Salyers, 2003; Raghunath, 2015). However, the relatively high percentages of *tdh* and *trh* positive *V. parahaemolyticus* isolates identified in food samples represent a potential public health risk.

Recently, the utility of molecular techniques has been established in epidemiological studies of *V. parahaemolyticus* infections and used for the analysis of genetic diversity. As a relatively simple method, ERIC-PCR was easier to perform than PFGE. It also was appropriate for the analysis of the large population of strains (Khan et al., 2002; Chen et al., 2012). Using this approach, the isolates were classified into five clusters when a 0.67 similarity cutoff value was used. However, we did not identify significant association between ERIC-PCR clusters and collection sites (source and provinces). Nevertheless, these result revealed high level of genetic diversity among *V. parahaemolyticus* isolates. Wong et al. (1999) showed a link between environment and likelihood of an outbreak (Mala et al., 2016). We also obtained similar results. MLST is also a useful method for typing strains because of its reproducibility and comparability. The approach has been commonly used for analysis of *V. parahaemolyticus* isolates (Guerrero et al., 2017). In this study, our isolates were grouped into five main clusters (I, II, III, IV, and V). MLST analysis revealed that the same source strains were in the same clusters (III, IV, and V) and suggested that these isolates have similar genotypes. As previously reported, the same ST types generally have the same serotype (Lüdeke et al., 2015). Multiple ST types were identified in our research. For example, ST291, ST300, and ST396 were identified in a public database as environmental isolates from China; ST1228, ST1251, and ST1231 were isolated from RTE foods; ST40 and ST162 were separated from environmental and clinical samples from the United States. There were also some clinical strains identified such as ST112 and ST739. ST212, which was isolated from clinical samples in Peru, showed greater evolutionary distance compared with other strains in our study. MLST analysis confirmed the genetic relatedness and genetic diversity occurring within those strains. Thus, ERIC-PCR and MLST are useful as phylogenetic research tools and as important methods for investigating outbreaks of *V. parahaemolyticus*.

Diarrhea due to the seafood borne pathogen *V. parahaemolyticus* has been a longstanding problem. In general, we provided the first comprehensive research on *V. parahaemolyticus* isolates from seafood in South China in both the summer and the winter by describing the prevalence, antibiotic resistance phenotypes, virulence, and molecular diversity. Our study showed that the prevalence of *V. parahaemolyticus* is 19.44%. The percentages of the isolates possessing *tdh* or *trh* were 8.16 and 12.24%, respectively. The antimicrobial-resistance patterns showed that ampicillin-resistance was widespread (79.59%). ERIC-PCR and MLST typing showed genetic relatedness and genetic diversity within these isolates. As seafood is a common and popular food choice in China, our findings should serve as a reference for understanding antibiotic-resistant trends in *V. parahaemolyticus* strains, providing information for evaluating this bacteria at the level of consumption, and establishing the appropriate monitoring programs, which are important in ensuring the safety of seafood.

## Author Contributions

YY finished the experiments and wrote the article. HY gave the idea and experimental support. JX and HL provided assistance and guidance throughout the research. ST and YC helped to finish the experiments. All authors assisted in writing the article and manuscript editing.

## Conflict of Interest Statement

The authors declare that the research was conducted in the absence of any commercial or financial relationships that could be construed as a potential conflict of interest.

## Footnotes

1 http://pubmlst.org/vparahaemolyticus/
