# Supplementary material for: Prevalence, Antibiotic Susceptibility and Diversity of Vibrio parahaemolyticus Isolates in Seafood from South China
Source: Front Microbiol. 2017 Dec 20;8:2566. doi: 10.3389/fmicb.2017.02566 (PMC5742333; doi:10.3389/fmicb.2017.02566)
Supplement: Supplementary file 1 [file Table_1.DOCX]

**Supplementary Materials**

**Table S1. Results of antimicrobial resistance, virulence genes, and MLST of V. parahaemolyticus isolates in this study**

| NO. | Source | Location | Resistance profileb | *toxR* | *tdh* | *trh* | *dnaE* | *gyrB* | *recA* | *dtdS* | *pntA* | *pyrC* | *tnaA* | MLST |
| --- | --- | --- | --- | --- | --- | --- | --- | --- | --- | --- | --- | --- | --- | --- |
| Vps01 | Fish | Guangdong | AMP-K-TE | + | - | - | 51 | 4 | 77 | 67 | 213 | 8 | 24 | 1228 |
| Vps02 | Fish | Guangdong | AMP-PRL-CN-K-S | + | - | - | 5 | 25 | 19 | 45 | 4 | 11 | 24 | 74 |
| Vps03 | Fish | Guangdong | AMP-PRL | + | - | - | 35 | 50 | 63 | 27 | 49 | 46 | 26 | 154 |
| Vps04 | Oyster | Guangdong | AMP-KZ-S-TE | + | - | - | 28 | 17 | 21 | 79 | 20 | 23 | 24 | 161 |
| Vps05 | Oyster | Guangdong | KZ-CN-K-S | + | - | - | 232 | 56 | 229 | 23 | 56 | 11 | 36 | 707 |
| Vps06 | Oyster | Guangdong | AMP-CN | + | - | - | 69 | 92 | 69 | 114 | 54 | 71 | 24 | 212 |
| Vps07 | Oyster | Guangdong | AMP-KZ-S | + | - | - | 6 | 274 | 77 | 229 | 46 | 211 | 150 | 555 |
| Vps08 | Oyster | Guangdong | AMP-K-S-TE | + | + | - | 148 | 355 | 74 | 103 | 127 | 101 | 202 | 1239 |
| Vps09 | Oyster | Guangdong | AMP-CN-K | + | - | - | 5 | 4 | 19 | 45 | 4 | 11 | 24 | 85 |
| Vps10 | Oyster | Guangdong | KZ-S-CIP-SXT | + | - | - | 10 | 106 | 31 | 18 | 23 | 150 | 26 | 821 |
| Vps11 | Oyster | Guangdong | K-S-SXT | + | - | - | 142 | 29 | 10 | 7 | 4 | 24 | 20 | 313 |
| Vps12 | Oyster | Guangdong | S | + | - | - | 44 | 130 | 87 | 110 | 29 | 37 | 63 | 193 |
| Vps13 | Shrimp | Guangdong | AMP-KZ-K-S | + | - | - | 71 | 13 | 67 | 77 | 21 | 69 | 55 | 158 |
| Vps14 | Shrimp | Guangdong |  | + | - | - | 51 | 4 | 77 | 67 | 213 | 8 | 24 | 1228 |
| Vps15 | Shrimp | Guangdong | AMP-PRL-KZ-K-S-TE-CIP | + | - | - | 3 | 4 | 19 | 4 | 29 | 22 | 22 | 227 |
| Vps16 | Shrimp | Guangdong | AMP-K | + | - | - | 35 | 20 | 31 | 29 | 23 | 6 | 27 | 58 |
| Vps17 | Shrimp | Guangdong | AMP-PRL-CN-SXT | + | - | - | 158 | 261 | 226 | 299 | 2 | 192 | 54 | 1019 |
| Vps18 | Shrimp | Guangdong |  | + | - | + | 69 | 92 | 69 | 114 | 54 | 71 | 24 | 212 |
| Vps19 | Fish | Hainan | AMP-KZ-K | + | - | - | 171 | 222 | 113 | 126 | 4 | 62 | 23 | 428 |
| Vps20 | Fish | Hainan | KZ-S-TE | + | - | - | 235 | 22 | 25 | 273 | 164 | 254 | 20 | 734 |
| Vps21 | Fish | Hainan | AMP-KZ-CN | + | + | - | 14 | 30 | 67 | 46 | 27 | 11 | 13 | 300 |
| Vps22 | Fish | Hainan | AMP-K | + | + | - | 234 | 285 | 230 | 278 | 31 | 177 | 57 | 699 |
| Vps23 | Fish | Hainan | PRL-KZ | + | - | - | 22 | 28 | 33 | 13 | 8 | 19 | 14 | 41 |
| Vps24 | Fish | Hainan | AMP-PRL-K-S-TE | + | - | - | 35 | 87 | 142 | 159 | 31 | 51 | 73 | 335 |
| Vps25 | Oyster | Hainan | AMP-K-S | + | - | - | 233 | 75 | 64 | 67 | 55 | 252 | 50 | 714 |
| Vps26 | Oyster | Hainan | AMP-KZ-S | + | - | - | 3 | 29 | 98 | 67 | 26 | 121 | 33 | 277 |
| Vps27 | Oyster | Hainan | S-CIP | + | - | - | 14 | 30 | 67 | 46 | 27 | 11 | 13 | 300 |
| Vps28 | Oyster | Hainan | AMP-KZ-CN-SXT | + | - | - | 31 | 131 | 60 | 78 | 52 | 62 | 13 | 206 |
| Vps29 | Oyster | Hainan | K-S | + | - | - | 93 | 116 | 6 | 19 | 24 | 93 | 48 | 140 |
| Vps30 | Oyster | Hainan | KZ-K-S | + | - | - | 50 | 55 | 48 | 52 | 23 | 53 | 43 | 92 |
| Vps31 | Oyster | Hainan | LEV | + | - | - | 14 | 30 | 67 | 46 | 27 | 11 | 13 | 300 |
| Vps32 | Oyster | Hainan | AMP-K-S-TE | + | - | - | 26 | 16 | 13 | 24 | 31 | 33 | 2 | 47 |
| Vps33 | Oyster | Hainan | AMP-CN-K-S | + | - | - | 22 | 24 | 17 | 13 | 9 | 19 | 14 | 40 |
| Vps34 | Oyster | Hainan | AMP-KZ-CN-SXT | + | - | - | 50 | 55 | 48 | 52 | 23 | 53 | 43 | 92 |
| Vps35 | Shrimp | Hainan | AMP-PRL-S-CIP | + | - | - | 132 | 391 | 31 | 29 | 197 | 314 | 26 | 1034 |
| Vps36 | Shrimp | Hainan | AMP-K-S | + | - | + | 126 | 368 | 25 | 333 | 50 | 297 | 33 | 934 |
| Vps37 | Shrimp | Hainan | AMP-S-TE | + | - | - | 26 | 25 | 144 | 117 | 4 | 104 | 33 | 688 |
| Vps38 | Shrimp | Hainan | AMP-KZ-K-S-SXT | + | - | - | 12 | 178 | 25 | 153 | 27 | 78 | 54 | 308 |
| Vps39 | Shrimp | Hainan | AMP-KZ | + | - | - | 5 | 52 | 27 | 13 | 18 | 25 | 40 | 82 |
| Vps40 | Shrimp | Hainan | AMP-KZ-CN | + | - | - | 14 | 30 | 67 | 46 | 27 | 11 | 13 | 300 |
| Vps41 | Shrimp | Hainan | AMP-S | + | - | - | 31 | 121 | 60 | 66 | 52 | 62 | 23 | 186 |
| Vps42 | Shrimp | Hainan | AMP-CN-S-SXT | + | - | - | 112 | 132 | 201 | 13 | 4 | 238 | 206 | 913 |
| Vps43 | Shrimp | Hainan | AMP-K-S | + | - | + | 11 | 75 | 64 | 151 | 124 | 7 | 50 | 1235 |
| Vps44 | Shrimp | Fujian | AMP-K-S | + | - | - | 60 | 192 | 147 | 29 | 100 | 147 | 110 | 357 |
| Vps45 | Fish | Fujian | AMP-KZ-S | + | - | + | 60 | 170 | 133 | 145 | 2 | 130 | 26 | 291 |
| Vps46 | Fish | Fujian | AMP-KZ-K | + | - | - | 50 | 55 | 48 | 52 | 23 | 53 | 47 | 263 |
| Vps47 | Fish | Fujian | AMP-KZ-S | + | + | - | 148 | 355 | 74 | 19 | 127 | 101 | 202 | 847 |
| Vps48 | Oyster | Fujian | AMP-K | + | - | - | 148 | 355 | 74 | 19 | 127 | 101 | 202 | 847 |
| Vps49 | Oyster | Fujian | AMP-S-SXT | + | - | - | 132 | 16 | 286 | 371 | 26 | 76 | 54 | 1243 |
| Vps50 | Oyster | Fujian | AMP-S | + | - | - | 17 | 331 | 235 | 23 | 33 | 137 | 94 | 739 |
| Vps51 | Oyster | Fujian | CN-K-S | + | - | - | 60 | 170 | 133 | 145 | 2 | 130 | 26 | 291 |
| Vps52 | Oyster | Fujian | AMP-CN-K | + | - | - | 12 | 184 | 188 | 191 | 28 | 168 | 23 | 1028 |
| Vps53 | Oyster | Fujian | KZ-S | + | - | - | 82 | 85 | 78 | 19 | 60 | 69 | 24 | 170 |
| Vps54 | Oyster | Fujian | K | + | - | - | 208 | 300 | 213 | 29 | 147 | 46 | 24 | 859 |
| Vps55 | Oyster | Fujian | AMP-K-S | + | - | - | 222 | 128 | 21 | 69 | 46 | 236 | 12 | 631 |
| Vps56 | Oyster | Fujian | AMP-KZ-S | + | - | - | 116 | 149 | 72 | 76 | 45 | 62 | 26 | 247 |
| Vps57 | Oyster | Fujian | AMP-KZ-K-S | + | - | - | 97 | 127 | 101 | 29 | 78 | 99 | 47 | 194 |
| Vps58 | Shrimp | Fujian | AMP-S | + | - | - | 269 | 25 | 266 | 280 | 50 | 78 | 94 | 269 |
| Vps59 | Shrimp | Fujian | AMP-K-S | + | - | - | 60 | 170 | 133 | 145 | 2 | 130 | 26 | 291 |
| Vps60 | Shrimp | Fujian | AMP-S | + | - | - | 27 | 324 | 61 | 164 | 50 | 54 | 44 | 983 |
| Vps61 | Shrimp | Fujian | AMP- | + | - | - | 7 | 217 | 61 | 76 | 128 | 131 | 64 | 880 |
| Vps62 | Shrimp | Fujian | AMP-KZ-K-S | + | - | + | 19 | 4 | 88 | 2 | 34 | 18 | 23 | 62 |
| Vps63 | Fish | Jiangxi | AMP-KZ | + | - | - | 28 | 59 | 54 | 29 | 26 | 11 | 24 | 105 |
| Vps64 | Oyster | Jiangxi | KZ-CN-K-S | + | - | - | 9 | 213 | 165 | 185 | 2 | 46 | 1 | 396 |
| Vps65 | Oyster | Jiangxi | S | + | - | - | 44 | 260 | 31 | 67 | 26 | 200 | 99 | 1302 |
| Vps66 | Fish | Hunan | AMP-K-S | + | - | - | 41 | 40 | 36 | 41 | 36 | 39 | 32 | 68 |
| Vps67 | Shrimp | Hunan | AMP- | + | - | - | 9 | 213 | 165 | 185 | 2 | 46 | 1 | 396 |
| Vps68 | Shrimp | Hunan | AMP-KZ-K-S | + | - | - | 230 | 139 | 279 | 347 | 26 | 203 | 26 | 1032 |
| Vps69 | Shrimp | Hunan | AMP-CN | + | - | - | 224 | 90 | 201 | 19 | 4 | 238 | 68 | 637 |
| Vps70 | Fish | Guangxi |  | + | - | - | 9 | 213 | 165 | 185 | 2 | 46 | 1 | 396 |
| Vps71 | Fish | Guangxi | AMP-KZ-K-S | + | - | - | 119 | 151 | 197 | 79 | 4 | 205 | 105 | 537 |
| Vps72 | Oyster | Guangxi | AMP-KZ-S | + | - | + | 10 | 1 | 62 | 95 | 50 | 85 | 26 | 124 |
| Vps73 | Oyster | Guangxi | AMP-TE-CIP | + | - | - | 98 | 229 | 112 | 107 | 18 | 97 | 23 | 434 |
| Vps74 | Shrimp | Guangxi | AMP-S | + | - | + | 54 | 64 | 3 | 35 | 14 | 46 | 24 | 106 |
| Vps75 | Shrimp | Guizhou | AMP-K | + | - | - | 58 | 332 | 10 | 8 | 28 | 33 | 183 | 719 |
| Vps76 | Shrimp | Guizhou | AMP-CN-S | + | - | + | 47 | 47 | 4 | 46 | 39 | 43 | 35 | 75 |
| Vps77 | Shrimp | Guizhou | AMP-KZ-S | + | - | - | 105 | 156 | 123 | 127 | 19 | 13 | 47 | 554 |
| Vps78 | Shrimp | Guizhou | AMP-KZ | + | + | - | 53 | 61 | 56 | 8 | 4 | 11 | 19 | 99 |
| Vps79 | Shrimp | Guizhou | AMP-S-TE | + | - | - | 111 | 5 | 77 | 34 | 20 | 171 | 24 | 866 |
| Vps80 | Fish | Yunnan | AMP-S | + | - | + | 47 | 58 | 53 | 19 | 50 | 37 | 26 | 162 |
| Vps81 | Fish | Yunnan | AMP-S | + | - | - | 26 | 212 | 91 | 69 | 23 | 5 | 26 | 977 |
| Vps82 | Fish | Yunnan | AMP-KZ-S | + | - | + | 5 | 303 | 173 | 373 | 152 | 318 | 23 | 1251 |
| Vps83 | Oyster | Yunnan | AMP-S-TE | + | - | - | 45 | 60 | 55 | 59 | 14 | 57 | 7 | 97 |
| Vps84 | Shrimp | Yunnan | KZ-S | + | - | - | 51 | 29 | 77 | 13 | 60 | 8 | 33 | 356 |
| Vps85 | Shrimp | Yunnan | AMP-K-S-C | + | + | - | 47 | 58 | 53 | 19 | 50 | 37 | 26 | 162 |
| Vps86 | Shrimp | Yunnan | AMP-S | + | - | - | 86 | 300 | 17 | 55 | 12 | 54 | 86 | 729 |
| Vps87 | Shrimp | Yunnan | AMP-CN-S | + | + | - | 17 | 18 | 9 | 59 | 4 | 28 | 18 | 310 |
| Vps88 | Shrimp | Yunnan |  | + | - | - | 270 | 371 | 273 | 120 | 23 | 238 | 26 | 996 |
| Vps89 | Fish | Chongqing | AMP-K-S-TE | + | - | - | 31 | 106 | 135 | 74 | 26 | 212 | 54 | 657 |
| Vps90 | Shrimp | Chongqing | AMP-KZ-S-SXT | + | - | + | 303 | 25 | 246 | 185 | 31 | 252 | 73 | 1241 |
| Vps91 | Shrimp | Chongqing | AMP-KZ-S | + | + | - | 196 | 380 | 19 | 29 | 193 | 176 | 61 | 987 |
| Vps92 | Shrimp | Chongqing | AMP-KZ-K-S-TE | + | - | + | 42 | 157 | 59 | 128 | 6 | 113 | 57 | 256 |
| Vps93 | Shrimp | Chongqing | AMP-S-TE | + | - | - | 158 | 116 | 14 | 281 | 54 | 43 | 26 | 703 |
| Vps94 | Shrimp | Chongqing | AMP-KZ-S | + | - | - | 79 | 43 | 31 | 86 | 21 | 11 | 12 | 177 |
| Vps95 | Fish | Sichuan | AMP-PRL-S | + | - | - | 47 | 58 | 53 | 13 | 50 | 37 | 26 | 1231 |
| Vps96 | Shrimp | Sichuan | AMP-KZ-K-S-C | + | - | - | 3 | 56 | 51 | 58 | 14 | 29 | 44 | 112 |
| Vps97 | Shrimp | Sichuan | AMP | + | - | - | 43 | 41 | 31 | 42 | 37 | 40 | 33 | 69 |
| Vps98 | Shrimp | Sichuan | AMP-KZ-S | + | - | - | 257 | 34 | 81 | 19 | 23 | 117 | 98 | 853 |
